# How Does *Tremblaya princeps* Get Essential Proteins from Its Nested Partner *Moranella endobia* in the Mealybug *Planoccocus citri*?

**DOI:** 10.1371/journal.pone.0077307

**Published:** 2013-10-21

**Authors:** Sergio López-Madrigal, Séverine Balmand, Amparo Latorre, Abdelaziz Heddi, Andrés Moya, Rosario Gil

**Affiliations:** 1 Institut Cavanilles de Biodiversitat y Biologia Evolutiva, Universitat de València, Paterna (València), Spain; 2 UMR203 BF2I, Biologie Fonctionnelle Insectes et Interactions, INSA-Lyon, INRA, Villeurbanne, France; 3 Área de Genómica y Salud, Fundación para el Fomento de la Investigación Sanitaria y Biomédica de la Comunitat Valenciana FISABIO – Salud Pública, València, Spain; University of South Florida College of Medicine, United States of America

## Abstract

Many insects maintain intracellular mutualistic symbiosis with a wide range of bacteria which are considered essential for their survival (primary or P-endosymbiont) and typically suffer drastic genome degradation. Progressive loss of P-endosymbiont metabolic capabilities could lead to the recruitment of co-existent facultative endosymbiont (secondary or S-endosymbiont), thus adding more complexity to the symbiotic system. *Planococcus citri*, among other mealybug species, harbors an unconventional nested endosymbiotic system where every *Tremblaya princeps* cell (β-proteobacterium) harbors many *Moranella endobia* cells (γ-proteobacterium). In this system, *T. princeps* possess one of the smallest prokaryote genome known so far. This extreme genome reduction suggests the supply of many metabolites and essential gene products by *M. endobia*. Although sporadic cell lysis is plausible, the bacterial participation on the regulation of the predicted molecular exchange (at least to some extent) cannot be excluded. Although the comprehensive analysis of the protein translocation ability of *M. endobia* PCVAL rules out the existence of specific mechanisms for the exportation of proteins from *M. endobia* to *T. princeps*, immunolocation of two *M. endobia* proteins points towards a non-massive but controlled protein provision. We propose a sporadic pattern for the predicted protein exportation events, which could be putatively controlled by the host and/or mediated by local osmotic stress.

## Introduction

Symbiosis is a natural widespread phenomenon that has been postulated to be a key factor for the evolutionary success of insects, many of which maintain mutualistic symbiotic relationships with intracellular bacteria. These endosymbiotic bacteria inhabit specialized host cells (bacteriocytes) and complement their normally unbalanced diets [1], [2], [3]. According to their dispensability for insect survival, they are classified as primary (P) or obligate endosymbionts, and secondary (S) or facultative symbionts, respectively. Thus, while S-symbionts can be horizontally transferred, are not necessarily present in every individual of a certain host species and can be placed outside bacteriocytes [4], P-endosymbionts are only vertically transmitted (from mothers to offspring). Strong incidence of genetic drift, together with relaxation of purifying selection on genes rendered unnecessary in the intracellular environment, lead P-endosymbiont genomes to undergo a huge size reduction. Eventually, if an S-symbiont is present, interactions among both bacteria and the eukaryotic host would take place, and new genes will become redundant. Thus, the P-endosymbiont might lose genes involved in the provision of metabolic capabilities required by the host, which can still be recruited from the co-existing S-symbiont (then becoming co-primary) [5]. Ongoing degeneration of both bacterial genomes could eventually cause a reciprocal metabolic complementation, adding more complexity to this ecological system [6], [7], [8], [9].

Many studied mealybug species from the subfamily Pseudococcinae harbor an unusual nested endosymbiotic organization [10], [11] in which each cell of the β-proteobacteria *Candidatus* Tremblaya princeps (*T. princeps* from now on, for the sake of simplicity) harbors several cells of another endosymbiont belonging to different bacterial clades depending on the host species. Both members of the consortium seem to be closely involved in the nutritional and reproductive physiology of their hosts [12]. In the mealybug *Planococcus citri*, *T. princeps* harbors the γ-proteobacterium *Candidatus* Moranella endobia (*M. endobia* from now on). Although *T. princeps* was originally considered as the P-endosymbiont according to phylogenetic criteria [13], recent complete genome sequencing of the two endosymbionts from two *P. citri* strains (PCIT and PCVAL) showed that both bacteria are functionally co-primaries [14], [15], [16], and display an unprecedented level of metabolic complementation between them. *T. princeps* possess the second smallest prokaryote genome described so far, most of which is devoted to the production of nearly complete ribosomes, with almost null metabolic capabilities except for the assembly of [Fe-S] clusters and the ability to partially synthesize some essential amino acids. Not only a huge range of metabolites but also proteins and tRNAs are supposed to be transferred from *M. endobia* to *T. princeps* in order to perform even essential informational functions, i. e., replication, transcription and translation.

Despite all mentioned *in silico* predictions, the way *M. endobia* proteins are recruited by *T. princeps* remains unknown. A recent survey of the *P. citri* nuclear genome led to the discovery that several genes of bacterial origin (neither from *T. princeps* nor *M. endobia*), involved in peptidoglycan production and recycling, have been acquired by the host by horizontal gene transfer, and are being expressed in bacteriocytes [17]. The authors propose that these genes might be involved in the release of the *M. endobia* cytoplasmic content by cell lysis. However, the existence of controlled mechanisms for specific macromolecules exportation from *M. endobia* to *T. princeps* cannot be ruled out.

The Sec machinery is the most generally employed mechanism for protein translocation across the inner membrane in Gram-negative bacteria, including endosymbionts. Sec-dependent secretory proteins can be exported, periplasmic and outer membrane proteins. They are synthesized at the cell cytoplasm as precursor macromolecules, carrying cleavable amino-terminal signal peptide (SP) sequences. Although *M. endobia* encodes an apparently functional Sec translocation complex, proteins with SP appear to be scarce in its proteome. McCutcheon and von Dohlen [14] have roughly reported that only 27 proteins contain SP sequences in *M. endobia* PCIT. Nevertheless, protein exportation might still take place through an abnormally permeable Sec translocation complex. Alternatively, the proteins can be exported through a non-specific transport mechanism, such as the permissive MscL membrane channel. The *msc*L gene has been preserved in the *M. endobia* genome [14], [16] while it is absent in all other known endosymbionts with reduced genomes. MscL forms a mechanosensitive channel which acts as a pressure release valve allowing solutes to exit the cell through a large pore in response to environmental osmotic downshift [18], [19], [20], and passage of small macromolecules through it has been described [21], [22], [23].

In order to better understand the mechanisms behind the provision of essential *M. endobia* proteins to the *T. princeps* cytoplasm, we have explored *in silico* the potential of the *M. endobia* Sec translocon machinery to participate on the process, and applied inmunohistochemistry assays with polyclonal antibodies to reveal the location of two *M. endobia* proteins throughout the nested endosymbiotic system: the channel protein MscL, only encoded in the *M. endobia* genome, and the chaperone Hsp60 (GroEL), a highly expressed protein in endosymbionts [1] that is also encoded in the *T. princeps* genome. Our results show the lack of massive and constitutive protein traffic from *M. endobia* to the *T. princeps* cytoplasm. Thus, both *in silico* analysis and experimental evidences support a model were *M. endobia* proteins would mostly be retained in its cell, allowing the controlled passage of needed macromolecules and metabolites through a highly permissive cell wall, whose strength could be controlled by the insect host, and only sporadically releasing its cytoplasmic content by cell lysis.

## Results and Discussion

### The Sec Protein Secretion Pathway in *M. endobia*, under Scrutiny

The dramatic reduction of the *T. princeps* genome implies the need for the recruitment of a wide range of proteins, whose unique possible source is the *M. endobia* cytoplasm, for the performance of essential cellular functions [14], [15], [16]. Although a simplified but likely functional Sec translocon is encoded by the *M. endobia* genome [14], [16], its potential implication in both endosymbionts molecular communication has not been analyzed in detail.

#### Signal sequences prediction along *M. endobia* proteome

To determine if *M. endobia* proteins that are essential for *T. princeps* could be exported by this general secretion system, we performed a comprehensive scanning of the 411 coding sequences from *M. endobia* str. PCVAL, using the PRED-TAT [24] and SignalPv4.1 softwares [25]. A putative SP sequence was predicted for 33 proteins at least by one of the softwares (Table 1), a fairly similar number to what has been mentioned for strain PCIT [14]. In 18 cases, the same program also detected a SP sequence at the *E. coli* and/or *S. glossinidius* homolog, although such motif was not always described in the literature, based on the information available at EcoCyc v16.5 [26]. In some other cases, SP has only been predicted in the *M. endobia* homolog. Several such proteins are known to maintain strict cytoplasmic distribution in other bacteria. They could be false positives or may represent cases of the gain of SP mimicking sequences by traditionally non-secreted proteins and, therefore, reflect possible adaptations to the nested endosymbiosis. In any case, most proteins for which an SP sequence has been predicted are usually located in the cell envelope and, only a small amount of cytoplasmic proteins needed to perform essential functions have been identified in this analysis. Even more, the two ribosomal proteins detected are also encoded by the *T. princeps* genome. Therefore, it is not plausible that a canonical Sec translocon could be used for the provision of essential proteins to *T. princeps*.

**Table 1:** *M. endobia* proteins potentially harboring SP sequences.

Gene	Protein product	Cellular function	Programe	SP	CS	Eco	Sgl	Location
***skp***	Chaperone	DegP/Skp folding pathway	SignalP	1.30	[AQA-ND]	+	+	P, C
***degQ***	Serine endoprotease	DegP/Skp folding pathway	PRED-TAT	1.33	[ARA-RP]	+	+	P
***yraP***	Uncharacterized protein	DegP/Skp folding pathway	PRED-TAT	1.22	[VGA-MV]	+	+	P
***surA***	Chaperone	OMP biogenesis	SignalP	1.20	[TLA-MS]	+	+	P
***znuA***	High-affinity zinc uptakesystem protein	ABC transporter	PRED-TAT	1.36	[AQA-AL]	+	+	P
***lptA***	LPS export system protein,LptA subunit	ABC transporter	PRED-TAT SignalP	1.34	[ALA-LT]	+	+	P
***lptD***	LPS transport and assemblycomplex, LptD subunit	LPS transport and assembly	PRED-TAT SignalP	1.25	[ARA-AL]	+	+	IM, OM
***lptE***	LPS transport and assemblycomplex, LptE subunit	LPS transport and assembly	PRED-TAT	1.24	[ATA-AT]	+	+	IM, OM
***bamA***	Outer membrane proteinassembly factor	OMP biogenesis	PRED-TAT SignalP	1.19	[SRA-DE]	+	+	IM, OM
***bamD***	Lipoprotein	OMP biogenesis	PRED-TAT SignalP	1.18	[VLA-DC]	+	+	IM, OM
***ompF***	Outer membrane protein F	Pore	PRED-TAT SignalP	1.21	[ARA-TE]	+	+	IM, OM
***dsbB***	Disulfide bond formationprotein B	Protein modification	PRED-TAT	1.24	[AFA-LE]	−	−	IM
***minD***	Septumsite-determining protein	Cell shape and division	PRED-TAT	1.21	[SSA-SI]	+	−	IM
***ygbQ***	Hypothetical protein	Cell shape and division	PRED-TAT	1.18	[QYA-LW]	−	+	IM
***nuoA***	NADH-quinoneoxidoreductase subunit A	Electron transport	PRED-TAT	1.41	[AQA-RT]	−	−	IM
***nuoL***	NADH-quinoneoxidoreductase subunit L	Electron transport	PRED-TAT	1.26	[RWS-EN]	+*	+	IM
***htpX***	Protease	Poorly characterized	PRED-TAT	1.29	[IQS-SS]	+*	+	IM
***ybgT***	Hypothetical protein	Poorly characterized	PRED-TAT	1.23	[ALA-IE]	+	−	IM, OM
***lpoB***	Lipoprotein,penicillin-bindingprotein activator	Peptidoglycan biosynthesis	PRED-TAT	1.25	[PQA-NI]	+	+	IM, OM
***mltC***	Membrane-boundlytic mureintransglycosylase C	Peptidoglycan biosynthesis	PRED-TAT	1.23	[THG-KE]	+	+	IM, OM
***lpdA***	Dihydrolipoyl dehydrogenase	Pyruvate and TCA metabolism	PRED-TAT	1.22	[SAA-FR]	−	−	IM, C
***dapB***	Dihydrodipicolinate reductase	Amino acids biosynthesis	PRED-TAT	1.26	[IQA-VT]	−	−	C
***fabB***	3-oxoacyl-[acyl-carrier-protein]reductase	Fatty acid biosynthesis	PRED-TAT	1.22	[AIA-ET]	−	−	C
***fabG***	3-oxoacyl-[acyl-carrier-protein]reductase	Fatty acid biosynthesis	PRED-TAT	1.22	[AIA-ET]	−	−	C
***serC***	Phosphoserine aminotransferase	Vitamin B6 metabolism	PRED-TAT	1.22	[SQA-QQ]	−	−	C
***erpA***	Iron-sulfur cluster insertionprotein erpA	FeS clusters biosynthesis	PRED-TAT	1.25	[LIA-AE]	−	−	C
***trxB***	Thioredoxin reductase	Electron transport	PRED-TAT	1.28	[ARA-NL]	−	−	C
***argS***	Arginyl-tRNA synthetase	Translation	PRED-TAT	1.26	[CEA-QV]	−	−	C
***deaD***	Cold-shock DEAD box protein A	Translation	PRED-TAT	1.25	[LQA-LT]	−	−	C
***rplK***	50S ribosomal protein L11	Translation	PRED-TAT	1.29	[ALG-QQ]	−	−	C
***rplL***	50S ribosomal protein L7/L12	Translation	PRED-TAT	27.49	[AEA-AE]	−	−	C
***tusB***	tRNA 2-thiouridinesynthesizing protein	Translation	PRED-TAT	1.21	[RSA-QT]	−	−	C
***yjeE***	ATP-binding protein	Poorly characterized	PRED-TAT	1.23	[VAA-AC]	−	−	C

SP coordinates, the more likely cleavage site (CS) for each protein, detection in *E. coli* (Eco) and *S. glossinidius* (Sgl), and subcellular location are indicated. (C) cytoplasm; (IM) inner membrane; (OM) outer membrane; (P) periplasm. *SP not described in EcoCyc v16.5.

#### Permeability of the Sec translocon machinery in *M. endobia*

A variety of genetic and biochemical approaches led to the molecular characterization of the Sec translocon machinery in *E. coli* through the description of a group of dominant mutations allowing the exportation of signal sequence-defective precursors or even signal sequence-less proteins, collectively called *prl* alleles [27], [28] These mutations are able to expand the repertoire of secretory proteins. To date, well characterized *E. coli prl* phenotypes have been linked to mutations on a total of 34 codons at genes *sec*A (*prlD* mutants) [29], [30], *sec*E (*prlG* mutants), *sec*G (*prlH* mutants) [31] and *sec*Y (*prlA* mutants) [28]. In order to evaluate the Sec translocon permeability in *M. endobia*, we performed comparative analysis between the products of these orthologous genes in *M. endobia* and *E. coli*. Identity levels range from 89.5% for SecY, 77.4% for SecA, 64.3% for SecE to 49.1% for SecG (unambiguous alignment of 440, 839, 126 and 110 amino acids, respectively). Nevertheless, among the 34 amino acids involved in *prl* mutations in *E. coli*, the vast majority (30) was conserved in *M. endobia* (Table S1). The four observed amino acid substitutions might have a small impact in protein function, since amino acid polarity and molecular weight is almost unaffected. Additionally, the impact of some of the mutations detected in *E. coli* in two of the cases are not clear, because they have been found in double mutants, and the additional change detected has been linked to a *prl* phenotype by itself. This is the case of the A277E change in *sec*Y observed in mutant *prlA7*, and E148K change in *sec*A observed in mutant *prlD21*.

According to our results, no clear evidences for abnormal Sec translocon permeability are observable in *M. endobia*, suggesting that SP sequences would be still necessary for *M. endobia* proteins exportation across this machinery. However, since overexpression of translocation machinery components such as SecA had also been linked to *prl* phenotypes [29], and analyzed residues are probably just a fraction of those potentially relevant for proper functioning, we cannot rule out such a possibility. Experimental approaches should be necessary in order to get a more realistic view on the molecular communication between both nested endosymbiosis members through this general protein secretion system.

### Search for Non-specific Protein Efflux Mechanisms

Since only a short set of *M. endobia* membrane proteins were shown to harbor secretory SPs (Table 1), and no evident permeability tolerance is expected for its Sec translocon (Table S1), we decided to explore the potential relevance of passive and non-specific transport mechanisms, such as mechanosensitive channels. MscL is one of several mechanosensitive ion channels that have been characterized in *E. coli*. However, it is the only one detected in *M. endobia*. An extensive search in the genomic databases showed that even this one is absent in all insect endosymbionts analyzed to date, including *T. princeps*.

MscL is known to release osmotic metabolites and ions in response of osmotic downshock, preventing cell lysis [18], [32]. It has also been empirically proven to allow the passage of small proteins up to 6.5 kDa [21], and there is some controversy regarding the possibility that thioredoxin (11.5 kDa) can go through it [21], [22]. Although other authors indicate that the 41-kDa chaperone DnaK [23], [33], the 52-kDa elongation factor Tu [23], [34], or even the 142-kDa enterobactin synthase EntF [35] can be released through MscL pores in osmotically stressed *E. coli* cells, the passage of these proteins through the channel has never been demonstrated, and it seems unlikely due to their large sizes [21]. However, it has been proven that *E. coli* cells subject to osmotic shock release up to 10% of their protein content, including periplasmic and cytoplasmic components under 100 kDa in a McsL-independent manner [36]. These authors suggest that osmotic shock might cause transient perforation of the plasma membrane, so that proteins can exit the damaged bacterial envelope pushed through a molecular sieve, whose limiting size is determined by the peptidoglycan mesh that surrounds the cell.

*In silico* predictions based on the *M. endobia* gene content and the discovery of several genes involved in peptidoglycan biosynthesis in the host nuclear genome indicate that the strength of the murein sacculus might be controlled by the host [17]. It is tempting to speculate that, if the above described mechanism is active in this bacterium, the peptidoglycan mesh could allow the passage of even larger proteins in a controlled manner. Furthermore, the outer membrane of *M. endobia* contains lipid IV_A_, lacking any secondary acyl chains and Kdo (2-keto 3-deoxy-D-manno-octulosonate), instead of the usual lipopolysaccharydes (LPS) typically observed in gram-negative bacteria. This particular composition could probably lead to a more fluid and permeable outer membrane [37], which might also facilitate the efflux of these large proteins. In this hypothetical scenario, the higher metabolic activity of *M. endobia*, compared with *T. princeps* would generate osmotic pressure on the *M. endobia* membrane, which could lead to the release of some cytoplasmic proteins through the unspecific mechanism above described until it is alleviated by the action of McsL. The channel could also participate in the extrusion of small peptides. Thus, the mechanism of protein passage would depend on the corresponding protein dimensions. Among the gene products that should be supplied by *M. endobia* to allow *T. princeps* functional viability, those involved in DNA replication and recombination, potentially responsible for the concerted evolution noticed on *T. princeps* genome [16], are especially relevant. As seen on Table 2, most of them are below 100 kDa of molecular weight and, therefore, could go through the *M. endobia* envelope by the proposed mechanism.

**Table 2:** *M. endobia* genes potentially involved in concerted evolution in *T. princeps,* ordered by their product sizes.

Gene	Product	PL(aa)	MW (kDa)
***priB***	Primosomal replication protein n	108	11.9
***ruvC***	Crossover junction endodeoxyribonuclease RuvC	164	17.9
***ssb***	Single-stranded DNA-binding protein	186	20.6
***ruvA***	Holliday junction ATP-dependent DNA helicase RuvA	206	22.9
***ruvB***	Holliday junction ATP-dependent DNA helicase RuvB	334	36.9
***recA***	Protein RecA	353	38.3
***recJ***	Single-stranded-DNA-specific exonuclease RecJ	579	63.1
***ligA***	DNA ligase	676	75.8
***dnaG***	DNA primase	582	66.6
***recG***	ATP-dependent DNA helicase RecG	695	78.1
***priA***	Primosomal protein N’	716	81.5
***polA***	DNA polymerase I	939	105.7
***recC***	Exodeoxyribonuclease V gamma chain	1101	127.0
***recB***	Exodeoxyribonuclease V beta chain	1181	134.6

PL: Predicted protein length; MW: Predicted protein molecular weight.

### Testing the Controlled Protein Efflux Mechanism *Versus* the Cell Lysis Hypotheses

Husnik and coworkers [17] suggested that the presence of expressed genes of bacterial origin involved in peptidoglycan biosynthesis and recycling in the host genome might be an indication that cell lysis is the mechanism used to provide *M. endobia* proteins and metabolites to *T. princeps*. If the above proposed scenario of controlled efflux of *M. endobia* proteins towards *T. princeps* were correct, only small quantities of *M. endobia* proteins would be present in *T. princeps* at a given time, contrary to what would be expected if they are released by cell lysis. In order to test this prediction, we performed experimental immunolocation studies to determine the spatial distribution of two proteins encoded by the *M. endobia* genome, GroEL and MscL, across the nested endosymbiotic system.

**Figure 1:**
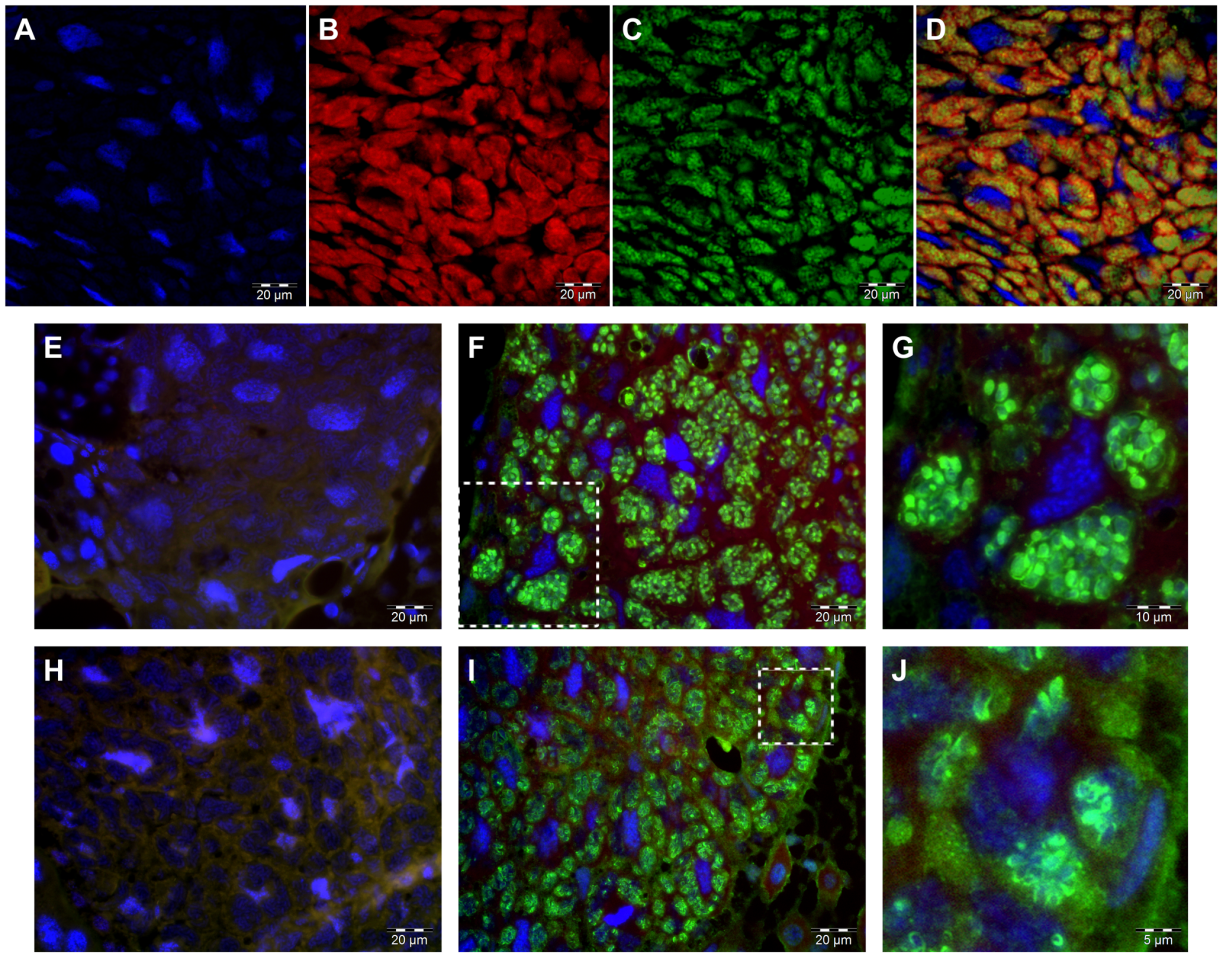
Microscopic analysis of *P. citri* PCVAL bacteriocytes showing the distribution of *M. endobia*’s GroEL and MscL. (**A–D**) FISH detection of *M. endobia* and *T. princeps* cells in *P. citri* bacteriocytes. Bacteriocyte nuclear genome is stained with DAPI (A, blue), *T. princeps* with probe b91 (B, red), *M. endobia* with probe g630 (C, green). Panel D shows the combination of all staining. (**E–J**) Immuno-histochemistry detection of GroEL (E–G) and MscL (H–J). Bacteriocyte nuclear genome is stained with DAPI (blue). Images G and J are amplifications of interesting details (in squares) from pictures F and H, respectively. No specific fluorescent signal was observed when serum of unimmunized rabbits was used (negative controls, E and H).

GroEL is an essential protein required for proper folding of a wide range of cytosolic proteins [38] It was selected because it has been proven to be highly expressed in endosymbiotic systems, where it is proposed to alleviate the destabilizing effects of non-synonimous mutations during protein folding [1], [39], [40]. In our experiments (Figure 1), fluorescent signal aggregated into bacilliform bodies (Figure 1, panels F, G), following a pattern that accurately mimics the one obtained when specific *M. endobia* detection is performed by Fluorescence In Situ Hybridization (FISH) experiments (Figure 1, panels C, D), thus proving that the protein is confined inside *M. endobia* cells. Only in a few cases, some GroEL staining was detected at the *T. princeps* inner membrane surface. Although there is also a *gro*EL gene in *T. princeps*, only the *M. endobia groEL* product could be detected. Even though GroEL is a highly conserved protein, this is not a completely unexpected result. First, the polyclonal antibodies used to detect the protein were generated against GroEL from *Buchnera aphidicola* APS [41], the endosymbiont of the pea aphid. Both *B. aphidicola* and *M. endobia* are γ-proteobacteria, whereas *T. princeps* is a β-proteobacteria. Additionally, differences between GroEL proteins from *M. endobia* and *T. princeps* are pronounced in terms of inmuno-histochemical detection, since most identical residues are located in short motives that are unlikely immunogenic (Figure S1). Thus, only half of the conserved residues map on potential minimal epitopes (5–8 residues), and only 38% of them map on amino acidic motives over 10 residues in size.

Location of MscL was approached using polyclonal antibodies specifically generated against the *M. endobia* protein. Again, the protein was only present in detectable levels in the *M. endobia* cells (Figure 1, panels I, J). In this case, and in coherence with its expected subcellular location, the shiner signal was recurrently detected only in *M. endobia* cell membranes. A few degenerating cells show some MscL staining in the *T. princeps* cytoplasm only, which suggests that the protein is not performing its functional role at the membrane in this bacterium. Thus, sporadic cell lysis might occur, but the pattern of distribution of both GroEL and MscL appears to indicate that there is not a massive supply of *M. endobia* proteins to the *T. princeps* cytoplasm, as it would be expected if such supply were accomplished mainly by cell lysis.

## Conclusions

The results of immuno-histochemical assays do not provide any evidence supporting constitutive and massive supply of *M. endobia* proteins to *T. princeps*. On the contrary, they indicate that (at least) the tested proteins accumulate only in *M. endobia* cells. A comprehensive scanning of *M. endobia* proteins that could potentially be targeted for exportation through the conserved Sec translocon machinery did not explain the intense protein traffic predicted to occur between both endosymbiotic bacteria. Neither putatively exported-protein adaptations, nor already described changes allowing abnormal permeability of the Sec translocon machinery for proteins without SP could be found. Although controlled and/or sporadic cell lysis can occur, the role of passive and non-specific communication gates, such as transient perforation of the plasma membrane and membrane MscL channels, both controlled by osmotic stress, can provide alternative explanations for the *M. endobia*-*T. princeps* molecular communication. Putatively recurrent osmotic stress events could result from the extremely biased distribution of metabolic capabilities between both endosymbionts. The unusual composition of the outer membrane lipopolysaccharides, in combination with the modulation of the strength of the peptidoglycan mesh controlled by the host, might also help on the provision of essential proteins from *M. endobia* to the *T. princeps* cytoplasm.

## Materials and Methods

### Insects Collection and Sample Preparation

Adult females were sampled from a laboratory population of *P. citri* which was reared on pumpkins at room temperature. Insects were decapitated and placed into 4% paraformaldehyde for fixation. Samples were stored at 4°C in phosphate buffer saline (PBS) with 0.05% azide until their preparation for paraffin inclusion. To do so, samples were dehydrated through a graded ethanol series, from ethanol 70° to absolute ethanol, and washed twice in butanol at room temperature for 30 minutes. Then, they were embedded in paraffin and cut on a microtome at 3–5 µm thick sections that were placed on poly-lysine coated slides, air dried and kept at 4°C until experiments performance. Prior usage, paraffin sections were dewaxed in two methylcyclohexan baths followed by two absolute ethanol baths of 10 minutes each.

### Fluorescence In Situ Hybridization (FISH), Immunostaining and Microscopy

Samples devoted to FISH were coated with a few drops of 70% acetic acid while incubated in a 60°C hotplate during 1 minute, in order to permeabilize cellular membranes. Once rinsed with PBS, they were dehydrated again through a graded ethanol series and air-dried. Then, samples were incubated in hydrochloride acid 0.01N with pepsin 0.1 mg/ml during 10 minutes in a 37°C waterbath for deproteinization, rinsed again with PBS, and dehydrated in ethanol. Prehybridization was performed for 30 minutes at 45°C in a buffer composed of 79% hybridization buffer (NaCl 0.9M; Tris 20 mM; EDTA 5 mM, in water, pH 7.2), 20% Denhardt solution (Ficoll 5 g; PVP 5 g; Bovine Serum Albumin 5 g in 500 ml water) and 1% SDS. Sections were subsequently coated by 100 µl of prehybridization buffer plus 1 µg of the corresponding labeled probe and incubated 3 hours at 45°C. *T. princeps* and *M. endobia* specific detection was performed with probe b91 (5′-GCCTTAGCCCGTGCTGCCGTAC-3′, TAMRA labeled) and probe g630 (5′- CGAGACTCTAGCCTATCAGTTTC-3′, 6FAM labeled), respectively [10]. In order to preserve fluorescent signal, slides were kept in dark from this point on. Then, they were rinsed twice in PBS with SDS 0.1% at 45°C, and at room temperature in PBS and water. Once completely air-dried, slides were mounted with an aqueous mounting media made of Gel Mount and 3 ug/ml DAPI.

Samples devoted to immunostaining were rehydrated though an ethanol gradient to PBS, permeabilized with Triton-100 0.1% for 10 minutes at room temperature, washed with PBS, and incubated with 1% bovine serum albumin (BSA) in PBS during 30 minutes prior to primary antibody incubation at 4°C overnight. Detection of GroEL was performed with rabbit polyclonal serum raised against the *B. aphidicola* APS homologous protein [41]. A rabbit polyclonal serum against MscL *M. endobia* was generated by Covalab (Villeurbanne, France), using the peptides C-KQFSWVLKPAQGNR (amino acids 55–68) and C-HNKEEEETPNELSKQS (amino acids 103–118) that correspond to a periplasmic and a cytoplasmic epitope respectively, according to an alignment with the *E. coli* homolog protein [42]. MscL antiserum was immunopurified using an agarose column coupled with the peptides used for immunization. Unimmunized rabbit serum was used as negative control. Antisera were diluted at 1∶500 (GroEL) or 1∶200 (MscL) in PBS with 0.1% BSA for primary antiserum incubation. Then, sections were washed with PBS containing 0.2% Tween, and incubated for 1 hour with donkey anti-rabbit secondary antibodies (1∶600) labeled with Alexa fluor 488 for primary antibodies detection. In order to preserve secondary antibody fluorescence, subsequent PBS-Tween washing and rinses with both PBS and tap water were conducted in darkness. The samples were completely air-dried and mounted as previously described for FISH experiments.

Both FISH and immunostaining slides were observed under an epifluorescence microscope (Olympus IX81) using filters HQ535/50, D470/40 and HQ610/75 for green (FISH with probe g630 and immunostaining), blue (DAPI) and red (FISH with probe b91) signals. Cell F Software (AnalySIS) was used for image capturing and processing.

### Sequence Data Analysis

SP screening was performed using both PRED-TAT [24] and SignalPv4.1 [25] softwares. *M. endobia* PCVAL complete proteome (CP003881) was scanned in order to detect SP motives at N-terminal region of its 411 predicted CDS. In order to increase prediction accuracy, *E. coli* and *S. glossinidius* homologs to *M. endobia* candidate proteins were also scanned with both softwares. SP predictions were additionally contrasted with information on the corresponding *E. coli* proteins available at EcoCyc v16.5 http://ecocyc.org/ [26].

Protein molecular weights were estimated with the Compute pI/Mw tool from ExPASy (http://web.expasy.org/compute_pi/) [43].

Pairwise alignments were performed by ClustalW [44].

## Supporting Information

Figure S1 **Distribution of both** ***T. princeps*** **and** ***M. endobia*** **GroEL identical residues.** Conservation clusters with variable sizes along GroEL alignment were taken into account in order to evaluate protein similarities in terms of immuno-histochemistry detection. Identical orthologous residues got distributed among clusters with 1–6, 8, 9, 11, 15–18, 23 and 30 residues in length.
(DOCX)

Table S1 **Analysis of known critical residues at proteins of the Sec translocon** **in** ***M. endobia*** **.** Well characterized mutational changes known to yield *prl* phenotypes in *E. coli* have been considered. Studied residues are ordered according to their position in the corresponding proteins, from N to C-terminus. Superindex denotes co-existing mutations in double mutant strains (*prlA4*, *6*, *7* and *11* for SecY, and *prlD21* for SecA) and the deletion of two adjacent codons (*prlG8* for SecE).
(DOCX)
