# Supplementary material for: How Does Tremblaya princeps Get Essential Proteins from Its Nested Partner Moranella endobia in the Mealybug Planoccocus citri?
Source: PLoS One. 2013 Oct 21;8(10):e77307. doi: 10.1371/journal.pone.0077307 (PMC3804617; doi:10.1371/journal.pone.0077307)
Supplement: Table S1 — Analysis of known critical residues at proteins of the Sec translocon in M. endobia . Well characterized mutational changes known to yield prl phenotypes in E. coli have been considered. Studied residues are ordered according to their position in the corresponding proteins, from N to C-terminus. Superindex denotes co-existing mutations in double mutant strains (prlA4, 6, 7 and 11 for SecY, and prlD21 for SecA) and the deletion of two adjacent codons (prlG8 for SecE). (DOCX) [file pone.0077307.s002.docx]

| **Gene Product** | **Amino acid** | **Mutant alleles** | **Mutation details** | ***M. endobia* status** | ***E. coli* codon** | ***M. endobia* codon** |
| --- | --- | --- | --- | --- | --- | --- |
| **SecY** | 37 | *prlA8911* | Ser → Phe | 38Ser | TCT | TCG |
|  | 64 | *prlA300* | Phe → Cys | 65Phe | TTT | TTT |
|  | 65 | *prlA8914* | Asn → Tyr | 66Asn | AAC | AAT |
|  | 67 | *prlA3* | Phe → Cys | 68Phe | TTC | TTC |
|  |  | *prlA666* | Phe → Ser |  |  |  |
|  | 68 | *prlA726* | Ser → Pro | 69Ser | TCT | TCT |
|  |  | *prlA799* | Ser → Leu |  |  |  |
|  |  | *prlA8913* | Ser → Phe |  |  |  |
|  | 69 | *prlA9* | Gly → Asp | 70Gly | GGT | GGT |
|  |  | *prlA205* | Gly → Cys |  |  |  |
|  | 71 | *prlA302* | Ala → Asp | 72Ala | GCT | GCA |
|  | 73 | *prlA306* | ΔSer | 74Ser | AGC | AGC |
|  | 90 | *prlA304* | Ile → Asn | 91Ile | ATC | ATC |
|  | 188 | *prlA6* | Ser → Leu^a^ | 189Ser | TCA | TCC |
|  | 191 | *prlA200* | Ile → Ser | 192Ile | ATC | ATT |
|  | 274 | *prlA1* | Val → Gly | 276Val | GTA | GTT |
|  | 277 | *prlA7* | Ala → Glu^b^ | 279Thr | GCA | ACA |
|  | 278 | *prlA202* | Ile → Ser | 280Ile | ATC | ATT |
|  |  | *prlA208* | Ile → Asn |  |  |  |
|  |  | *prlA303* | Ile → Thr |  |  |  |
|  | 282 | *prlA401* | Ser → Arg | 284Ser | AGT | AGC |
|  | 286 | *prlA4* | Phe → Tyr^c^ | 288Phe | TTC | TTT |
|  | 407 | *prlA7, 11* and *301* | Leu → Arg^b,d^ | 409Leu | CTT | TTG |
|  | 408 | *prlA4* and *6* | Ile → Asn^a,c^ | 410Ile | ATC | ATA |
|  | 411 | *prlA11* | Val → Gly^d^ | 413Val | GTC | GTG |
| **SecE** | 105 | *prlG2* | Ser → Pro | 106Ser | TCA | TCA |
|  | 108 | *prlG1* | Leu → Arg | 109Leu | CTG | CTG |
|  | 116 | *prlG8* | ΔVal^e^ | 117Val | GTT | GTT |
|  | 117 | *prlG8* | ΔArg^e^ | 118Arg | CGC | CGT |
|  | 120 | *prlG3* | Ser → Phe | 121Ser | TCC | TCA |
| **SecG** | 25 | *prlH2* | Gly → Asp | 25Gly | GGT | GGT |
|  | 26 | *prlH1* | Lys → Glu | 26Lys | AAA | AAA |
|  | 50 | *prlH3* | Asn → Tyr | 50Thr | AAC | ACT |
|  |  | *prlH4* | Asn → Ile |  |  |  |
|  | 57 | *prlH6* | Ala → Glu | 57Ala | GCG | GCA |
|  | 65 | *prlH5* | Ile → Thr | 65Val | ATC | GTG |
| **SecA** | 111 | *prlD4* | Thr → Asn | 111Thr | ACC | ACT |
|  | 134 | *prlD22* | Tyr → Cys | 134Tyr | TAC | TAC |
|  |  | *azi-17* | Tyr → Asn |  |  |  |
|  |  | *prlD21* and *23* | Tyr → Ser^f^ |  |  |  |
|  | 148 | *prlD21* | Glu → Lys^f^ | 148Asp | GAA | GAT |
|  | 373 | *prlD5* | Ala → Val | 373Ala | GCT | GCG |
|  | 484 | *prlD43* | His → Gln | 484His | CAC | CAC |
|  | 488 | *prlD2* and *3* | Ala → Val | 488Ala | GCG | GCA |
|  | 507 | *prlD20, 24, 26-28* and *31* | Ala → Val | 507Ala | GGC | GGC |
|  | 645 | *azi-4* | Leu → Gln | 645Leu | CTG | CTA |

^a^Mutations in double mutant *prl*A6; ^b^Mutations in double mutant *prl*A7; ^c^Mutations in double mutant *prl*A4; ^d^Mutations in double mutant *prl*A11; ^e^Mutations in *prl*G8; ^f^Mutations in double mutant *prl*D21.
