# Supplementary material for: How Does Tremblaya princeps Get Essential Proteins from Its Nested Partner Moranella endobia in the Mealybug Planoccocus citri?
Source: PLoS One. 2013 Oct 21;8(10):e77307. doi: 10.1371/journal.pone.0077307 (PMC3804617; doi:10.1371/journal.pone.0077307)
Supplement: Supplementary file 2 [file pone.0077307.s001.docx]

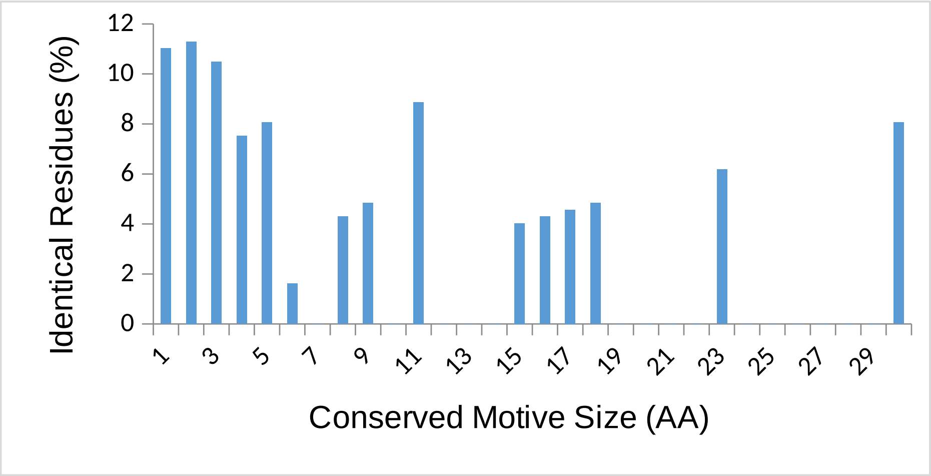
Identical Residues (%)
0
2
4
6
8
10
12
1
3
5
7
9
11
13
15
17
19
21
23
25
27
29
Conserved Motive Size (AA)
